# The efficacy of Kangaroo-Mother care to the clinical outcomes of LBW and premature infants in the first 28 days: A meta-analysis of randomized clinical trials

**DOI:** 10.3389/fped.2023.1067183

**Published:** 2023-02-27

**Authors:** Zhen Zhu, Xinchen Wang, Wenzeng Chen, Shuping Pei, Qingmin Wang, Hailian Guan, Guang Zhu

**Affiliations:** ^1^ Department of Obstetrics, Tongde Hospital of Zhejiang Province, Hangzhou, China; ^2^ Department of Gynaecology, Tongde Hospital of Zhejiang Province, Hangzhou, China

**Keywords:** Kangaroo-Mother care (KMC), efficacy, low birthweight (LBW) infants, premature infants, meta-analysis

## Abstract

**Objective:**

The objective of this study was to systematically determine the benefits of Kangaroo-Mother Care (KMC) on the clinical outcomes of low birthweight (LBW) and preterm infants.

**Methods:**

For this study, the following databases were retrieved for articles published until November 2021: PubMed, Web of Science, EBSCO, and the Cochrane library. The primary clinical outcome was mortality between enrollment and 28 days. The secondary clinical outcomes were the mean duration of hospital stay, hypothermia, sepsis, exclusive breastfeeding at the end of the neonatal period, and exclusive breastfeeding at discharge.

**Results:**

We conducted a meta-analysis, which included 17 RCTs, involving overall 17,668 participants. The results of this meta-analysis showed that KMC could reduce the primary clinical outcome of mortality between enrollment and 28 days (RR: 0.80, 95% Cl: 0.71–0.91, *p *< 0.01). For the secondary clinical outcomes, KMC had a varying degree of benefits on the mean duration of hospital stay (SMD: −0.96, 95% Cl: −1.02–0.90, *p *< 0.001), hypothermia (RR: 0.45, 95% Cl: 0.27–0.75, *p *< 0.01), and sepsis (RR: 0.79, 95% Cl: 0.70–0.89, *p *< 0.001). The exclusive breastfeeding at the end of the neonatal period and exclusive breastfeeding at discharge of KMC had benefits, which was not statistically different though (OR: 2.16, 95% Cl: 0.55–8.41, *p *= 0.27; OR: 1.16, 95% Cl: 0.82–1.64, *p *= 0.39, respectively).

**Conclusions:**

KMC was decreased mortality in LBW and premature infants between enrollment and 28 days. In addition, KMC also had a favorable effectiveness on the secondary clinical outcomes, such as mean duration of hospital stay, hypothermia, sepsis. Moreover, KMC also had a slight effectiveness on exclusive breastfeeding at the end of the neonatal period and exclusive breastfeeding at discharge.

## Introduction

Kangaroo Mother Care (KMC), originally defined as skin-to-skin contact between mother and newborn, frequent exclusive or almost exclusive breastfeeding, and early discharge, has been proposed as an alternative to traditional interventions of care for low birthweight (LBW) infants (1, 2) and is a multifaceted intervention for LBW infants, preterm infants and their parents (3). In the early 1970s, researchers studied the impact of extra contact between mothers and babies in the early stages of life. This entailed skin-to-skin contact with the mother's bare chest as quickly as possible after birth (4). This became known as “Kangaroo Care” (KC) (4). KC, is known as KMC or skin-to-skin contact (4). As a human-centered care intervention (5), KMC is effective in improving the survival of LBW and premature infants (6), whether KMC is initiated after the LBW infant's vital signs (34038632) are stabilized or is received prior to stabilization (2, 6). WHO has recommended that health facilities use KMC for LBW infants for more than a decade, and some studies have shown that in low- and middle-income countries, KMC for all LBW infants, regardless of birthplace, can significantly reduce neonatal mortality (7, 8). In addition, Charpak et al. found that KMC still had significant, enduring social and behavioral protective effects 20 years after the intervention, indicating that KMC was effective in promoting neurological development in newborns (3). KMC has many benefits not only for the newborns, but also for the mothers. In low- and middle-income countries, approximately 1 in 5 women experience postpartum depression, and mothers of LBW infants are at higher risk (9). Through a randomized clinical trial, Sinha et al. discovered that KMC significantly reduced the risk of moderate to severe depressive symptoms in the postpartum period (9). Based on this, this study attempted to evaluate the benefits of KMC systematically and intuitively from the effectiveness of KMC on the clinical outcomes (mortality, hospital stay, sepsis, hypothermia and exclusive breastfeeding) of LBW and preterm infants in the first 28 days, aiming to make it more widely used in clinical practice.

## Methods

### Search strategy

From its inception to November 6, 2021, PubMed, Web of Science, EBSCO, and Cochrane were searched to identify relevant research for this meta-analysis. Literature search was conducted combining the use of free-text terminology and medical subject headings. Search terms included “Kangaroo-Mother Care” or “Kangaroo Care”, and “low birthweight infant” or “the infant with low birthweight” or “LBW infant”, and “premature” or “premature infant.” In addition, we manually searched the references of included studies. This meta-analysis was instructed according to the Preferred Reporting Items for Systematic Review and Meta-Analysis Protocols (PRISMA-P) 2015 statement (10).

### Inclusion and exclusion criteria

Inclusion criteria were: (1) English reports were randomized controlled trials assessing the effectiveness of KMC on LBW and preterm infants; (2) studies compared KMC to standard care; (3) studies provided data on the clinical outcomes of LBW and preterm infants. Here, KMC is different from SSC. KMC is that the mother sits in a comfortable position wearing a loose top, the baby is held upright between the breasts with all limbs relaxed and their head turned to one side. During KMC, there is no feeding. Whereas SSC is that the dry, naked baby is lying prone on the mother's naked chest, usually covered with a warm blanket, from the inception of birth to the end of the first breastfeeding.

Exclusion criteria were: (1) participants were neither LBW nor premature infants; (2) single-arm studies or studies did not have suitable subgroup analysis; (3) either participants in the experimental group did not use KMC or participants in the control group did not use standard care; (4) research data could not be extracted. If there were continual publications or ongoing updates of studies, we would incorporate the latest study to ensure the credibility of this review.

### Clinical outcomes measure

The primary clinical outcome of this study was mortality between enrollment and 28 days. The secondary clinical outcomes were other relevant results (the mean duration of hospital stay, hypothermia, sepsis, exclusive breastfeeding at the end of the neonatal period, and exclusive breastfeeding at discharge).

### Assessment of the risk of bias and data extraction

The Cochrane Collaboration Risk of Bias Assessment tool (11) was used to assess the potential risk of bias in trials. Two investigators performed the review independently, and a third investigator resolved the disagreements. Baseline information included author, publication year, country, KMC vs. standard care, and number of participants; the primary clinical outcome included mortality between enrollment and 28 days; the secondary clinical outcomes included mean duration of hospital stay, hypothermia, sepsis, exclusive breastfeeding at the end of the neonatal period and exclusive breastfeeding at discharge. In addition, studies were included if they reported one or more clinical outcomes and not necessarily all.

### Statistical analysis

The primary clinical outcome and most secondary clinical outcomes were analysed by risk ratio (RR) and a 95% confidence interval (95% Cl). Among the secondary clinical outcomes, the mean duration of hospital stay was expressed by standardized mean difference (SMD) and a 95% Cl. This study selected the random-effects models when *I*^2^ was higher 50 and fixed-effects models when *I*^2^ was lower than or equal to 50. The Begg's and Egger's test was employed to evaluate publication bias, and *p *< 0.05 was considered statistically significant. The sensitivity analysis was performed using the Stata 12.0 Software (12).

## Results

### Eligible studies and inclusion characteristics

**Figure 1:**
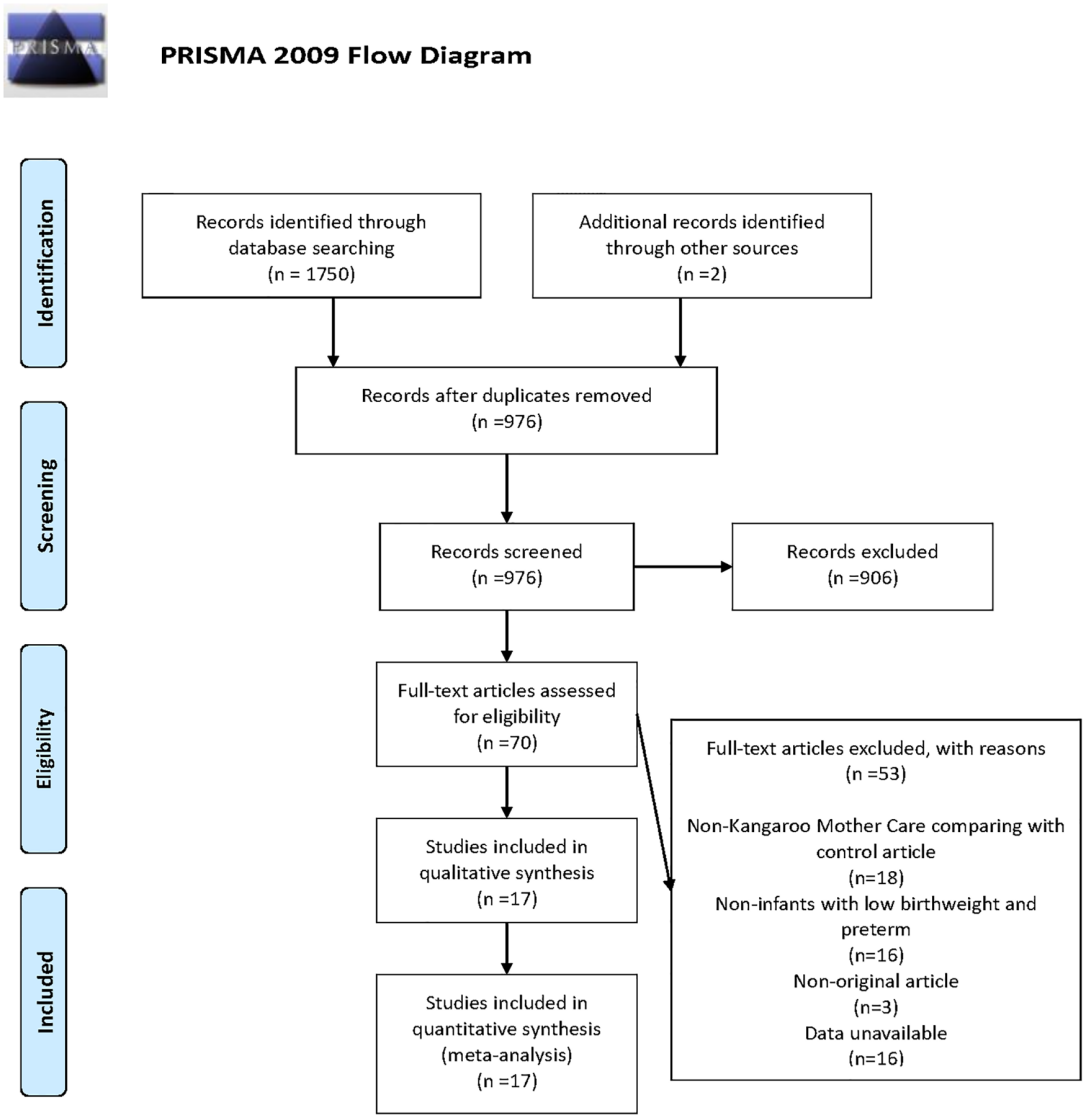
Flow diagram of study inclusion and exclusion.

According to the search strategy, 1,752 clinical trials from the database were preliminarily retrieved, including two manually retrieved studies. Due to the rigorous selection criteria, we excluded single-arm studies, studies where participants were not LBW or premature infants, or studies that did not fulfil all the inclusion criteria. Figure 1 shows the detailed search and screening process. In this study, 17 RCTs involving overall 17,668 participants were included, of which 14 studies (2, 13–15, 17, 19–26, 28) targeted at LBW infants and 3 studies (16, 18, 27) focused on premature infants. The quality of studies was detailed in Supplementary Table S2. Among the 17 studies, standard care used as the control group. The use of skin-to-skin contact was identified in one study (27). Apart from one study (16) where Kangaroo Care was applied, KMC was used in the remaining 15 studies (2, 13–15, 17–26, 28) among which one study (24) evaluated the efficacy of Community-Based Kangaroo Mother Care. As for the clinical outcomes of the newborns, 5 studies (2, 15, 21, 24, 28) mentioned the primary clinical outcome, and 15 studies (2, 13–23, 25–27) mentioned the secondary clinical outcomes, which were mean duration of hospital stay (*n* = 12), hypothermia (*n* = 7), sepsis (*n* = 4), exclusive breastfeeding at discharge (*n* = 3), exclusive breastfeeding at the end of neonatal period (*n* = 2), respectively. The characteristics of included studies were presented in Supplementary Table S1.

### The primary clinical outcome

**Figure 2:**
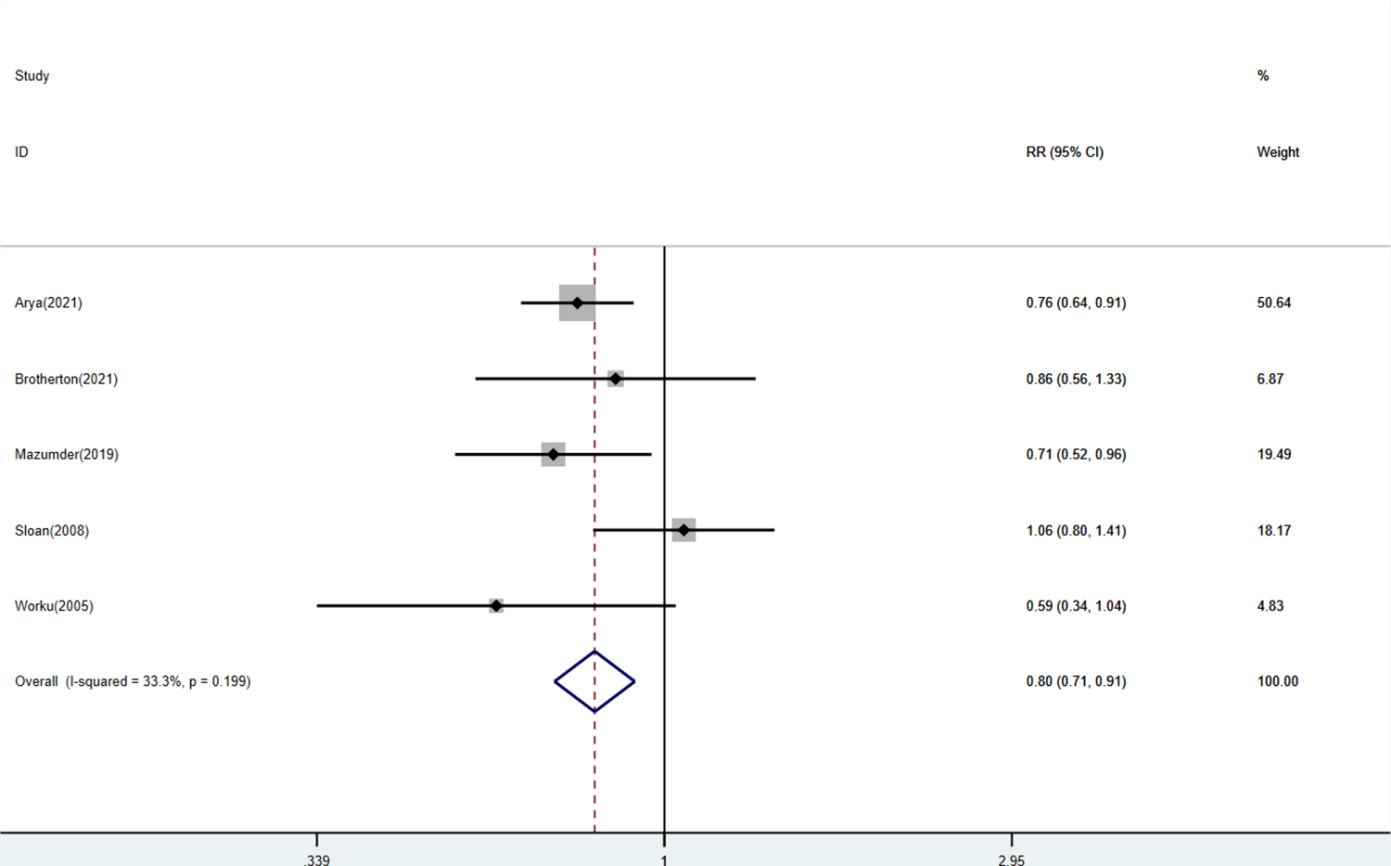
Forest plots of the mortality between enrollment and 28 days for KMC versus control group.

Among the included studies, five studies described mortality between enrollment and 28 days, including 16,162 neonates with 8,400 neonates in the experimental group and 7,762 neonates in the control group respectively. The comparison of KMC and the control group found that KMC could decrease the mortality of LBW and premature infants (RR: 0.80, 95% Cl: 0.71–0.91, *p *< 0.01; Figure 2) with a low heterogeneity.

### The secondary clinical outcomes

**Figure 3:**
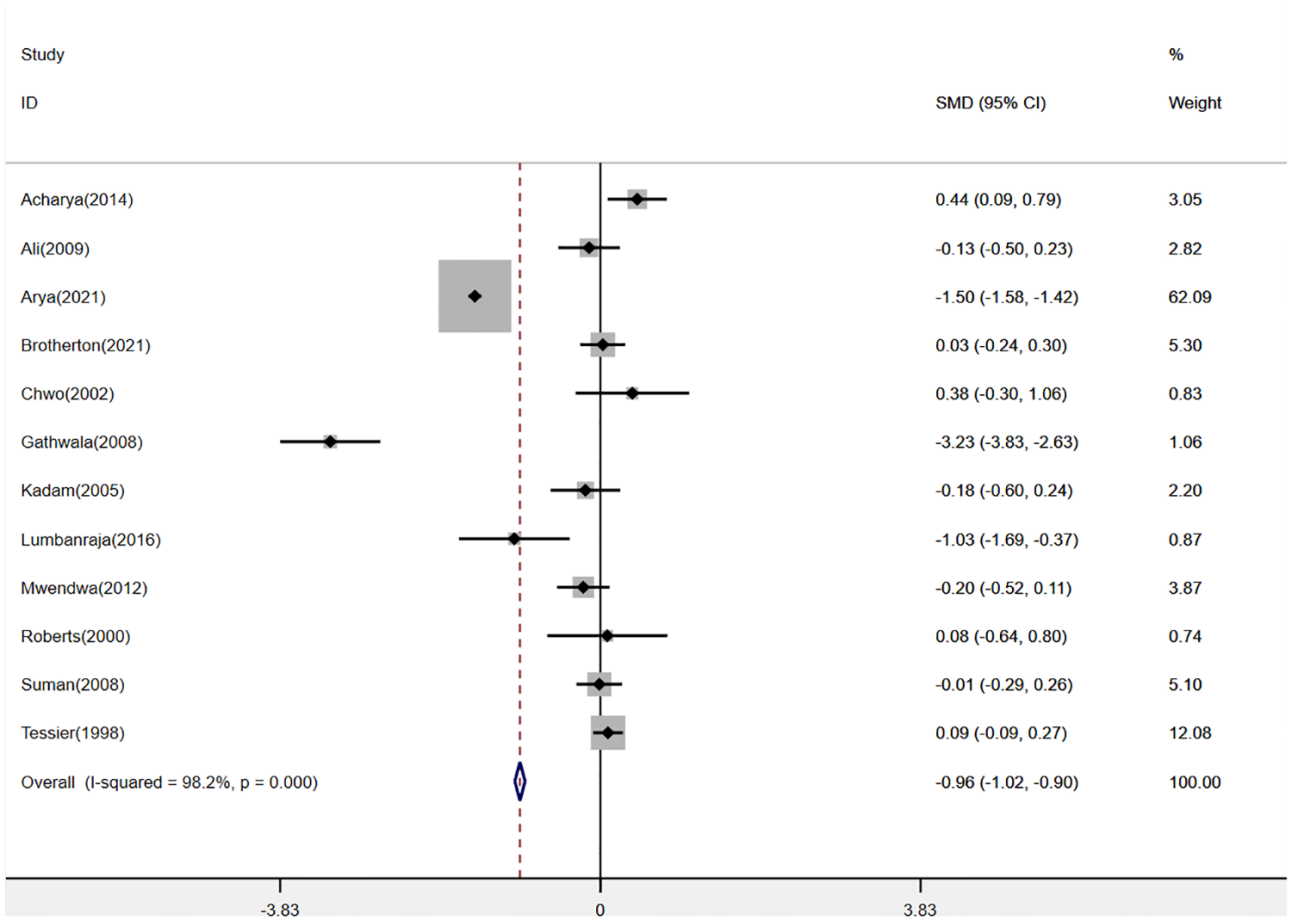
Forest plot of the mean duration of hospital stay for KMC versus control group.

**Figure 4:**
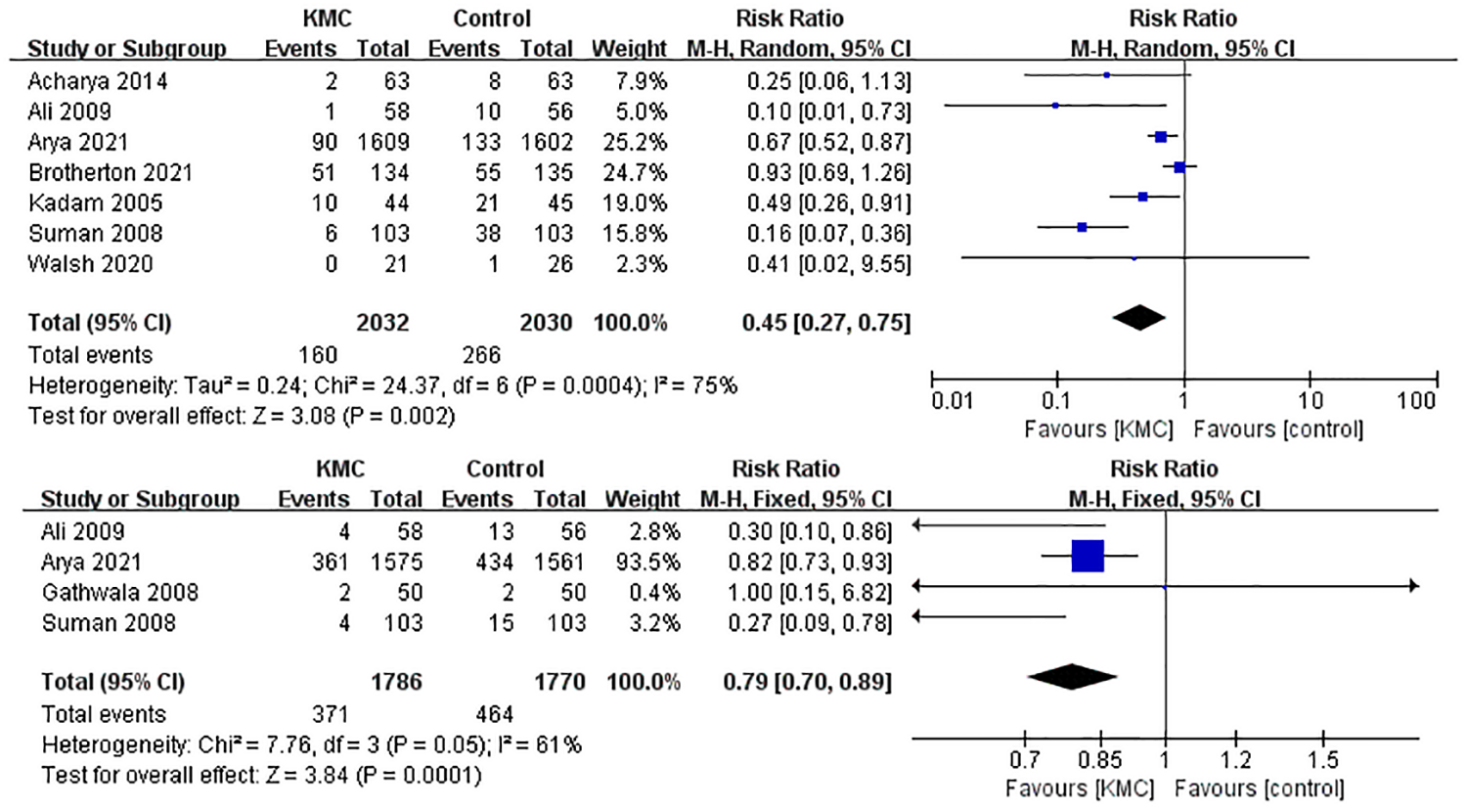
Forest plot of the hypothermia and sepsis for KMC versus control group.

Twelve studies (2, 14–17, 19, 20, 22, 23, 25–27) were pooled when analyzing the outcome of the mean duration of hospital stay. The analysis found that KMC could shorten the mean duration of hospital stay in LBW and premature infants (SMD: −0.96, 95% Cl: −1.02–0.90, *p *< 0.001, Figure 3). There was a total of 8 studies (2, 13–15, 17, 19, 25, 27) reporting hypothermia and sepsis. KMC could effectively reduce the incidence of hypothermia and sepsis (RR: 0.45, 95% Cl: 0.27–0.75, *p *< 0.01; RR: 0.79, 95% Cl: 0.70–0.89, *p *< 0.001; respectively, Figure 4). In addition, four studies (2, 15, 18, 21) mentioned exclusive breastfeeding at discharge and exclusive breastfeeding at the end of the neonatal period. There was a trend for KMC to increase exclusive breastfeeding at the end of the neonatal period and exclusive breastfeeding at discharge, however, this did not reach statistical significance (OR: 2.16, 95% Cl: 0.55–8.41, *p* = 0.27; OR: 1.16, 95% Cl: 0.82–1.64, *p* = 0.39, respectively, Supplementary Figure S1).

### Publication bias and sensitivity analysis of the secondary clinical outcomes

Given that we included 17 studies in this analysis, publication bias test was conducted to reassess the effectiveness of KMC. The results showed no publication bias, Egger's *p *= 0.135 and Begg's *p *= 0.881, both *p *> 0.05. Moreover, sensitivity analysis showed that the result remained consistent every time each study was removed consecutively (Supplementary Figures S2, S3).

## Discussion

From a general perspective, KMC was beneficial to the clinical outcomes (mortality, hospital stay, sepsis, hypothermia and exclusive breastfeeding) of LBW and premature infants in the first 28 days. Studies have shown that premature birth was the leading cause for morbidity and mortality in children under five years of age (29), and there were approximately 1 million children died of the complications of premature birth such as hypothermia each year (30). Hospital-based KMC could save the lives of LBW and premature infants (31). Salim et al. showed that KMC could reduce the mortality of newborns in the stable period (32), especially in sanitary environments with limited resources (31). KMC has been recommended by the World Health Organization for the care of LBW infants whose weight was 2,000 g or less (33). However, KMC has not been integrated into health systems worldwide (34), and the implementation of this method has been restricted (27). Further research found that medical care, time, social support, and family acceptance were the obstacles to the application of KMC (35). Therefore, this study attempted to evaluate the benefits of KMC systematically and intuitively centering around LBW and preterm infants to make it more widely applied in clinical practice.

This study showed that KMC could significantly reduce mortality between enrollment and 28 days compared with standard care. We speculated that it might be related to the position of KMC. Charpak et al. (5) have shown that the position of KMC (skin-to-skin contact to the mother's chest) could enhance the bond between the baby and its mother, increasing the baby's dependence on its mother (36), and give appropriate stimulation to protect against apnea events (37), meanwhile provide enough warmth (38) to prevent hypothermia-related mortality in premature infants (31). In addition, studies have shown that during KMC, the respiratory patterns of the parents could make babies more comfortable (39). Moreover, among premature infants, KMC could also increase weight (40), accelerate skin maturation (41), improve cerebral blood flow, affect brain structure and promote the development of the nervous system (42). Besides, babies fed by KMC had a lower heart rate (42). These positive effectiveness ensured that LBW and premature infants consumed less energy and were less stressed (43), thereby ensuring LBW and premature infants improved more rapidly. Garg et al. (44) pointed out that KMC was correlated with lower bilirubin levels in newborns, which may be explained by improved gastro-intestinal peristalsis during KMC. With increased defecation, the hepatoenteral circulation of bilirubin may decrease.

Moreover, parents of LBW or premature babies may also suffer psychologically since they were often not prepared for their babies' unexpected birth. By implementing KMC, nurses could help parents prepared and adapted to changes in their lives within a short of time and boost self-confidence (45). Some studies have shown that KMC could decrease mothers’ negative emotions (such as anxiety or depression) and promote positive parent–child interaction (46). We hypothesized that all the reasons mentioned above might explain the decreased mortality of LBW and premature infants.

For secondary clinical outcomes, KMC could shorten the median hospital stay of LBW and premature infants and reduce the incidence of hypothermia and sepsis. It might be related to the fact that KMC can promote the growth and development of LBW and premature infants (40). Studies have shown that KMC could effectively reduce the pain response during heel blood collection in premature infants, increase the length of sleeping time, and promote newborns' growth and development, thereby shortening the median length of hospital stay (43, 47). In addition, KMC was an effective and low-cost technique to prevent hypothermia in newborns (30). Since KMC could stabilize the heart and lungs through the autonomic nervous system (48) and promote the microcirculation, KMC could effectively increase the tissue temperature with increased blood flow among LBW and premature infants (49).

Interestingly, Hucklenbruch-Rother et al. found that KMC could significantly reduce the mRNA expression of six important stress response genes such as corticotropin-releasing hormone receptor 2 (CRH-R2), glucocorticoid receptor gene (NR3C1), and serotonin of the transporter gene (SLC6A4) in LBW and preterm infants, which affected the long-term expression of stress response related genes in premature infants (50). We speculated that the mechanism of action might explain the reason why KMC could reduce the incidence of sepsis. Nevertheless, the underlying molecular mechanism still needs to be further explored in depth.

In this meta-analysis, KMC could increase exclusive breastfeeding both at discharge and at the end of the neonatal period in LBW and premature infants to some extent. However, the difference did not reach statistical significance. We considered that the small number of included studies might be to blame. Yilmaz et al. showed that KMC could increase the self-efficacy of breastfeeding mothers and reduce the perceived insufficient milk supply, which indicated that KMC might have a great influence on breastfeeding perception (51). Furthermore, studies have also shown that the prolonged time of skin contact between mothers and newborns was related to the early initialization of exclusive breastfeeding (52). We hypothesized that it might be because the longer duration of skin contact yields better mother-infant interaction that significantly reduces the mother's emotional stress (53) and promotes the secretion of prolactin, thereby stimulating milk secretion. However, this hypothesis still needs to be further confirmed by future research.

From an overall perspective, this study has so far been the most comprehensive analysis of KMC on clinical outcomes of LBW and premature infants with a high level of evidence in that the included studies were all randomized controlled trials. Still, this study also had some shortcomings. This analysis mainly focused on outcomes that threat the newborns' health most and thus did not evaluate the effectiveness of KMC on other clinical outcomes such as weight gain, etc. Moreover, given the long time span of the included studies, there existed significant heterogeneity when analyzing secondary clinical outcomes. Hence, conclusions about secondary clinical outcomes in this study need to be further confirmed in the future.

## Conclusion

In summary, this study showed that KMC was beneficial to LBW and premature infants in any case, ranging from decreasing the incidence of hypothermia and sepsis, shortening the mean duration of hospital stay to reducing neonatal mortality. Therefore, KMC should be vigorously promoted, popularized and standardized in clinical practice (54). However, clinical outcomes of KMC involved in this study among LBW and preterm infants were not comprehensive enough. More in-depth and comprehensive randomized clinical experiments need to be conducted to better guide the clinical practice of KMC.

## Data Availability

The original contributions presented in the study are included in the article/**Supplementary Material**, further inquiries can be directed to the corresponding author.
