# Supplementary material for: The efficacy of Kangaroo-Mother care to the clinical outcomes of LBW and premature infants in the first 28 days: A meta-analysis of randomized clinical trials
Source: Front Pediatr. 2023 Feb 27;11:1067183. doi: 10.3389/fped.2023.1067183 (PMC10008937; doi:10.3389/fped.2023.1067183)
Supplement: Supplementary file 1 [file Table1.docx]

**Supplementary Table 1**. Characteristics of included clinical trials in the meta-analysis

| Author, year | Type of study | Therapeutic regimen | | Number of inclusions | | Population of study | Primary outcomes | Secondary outcomes |
| --- | --- | --- | --- | --- | --- | --- | --- | --- |
|  |  | Treatment | Control | Treatment | Control |  |  |  |
| Acharya, 2014 | RCT | KMC | Control | 63 | 63 | LBW infant |  | Mean duration of hospital stay,  Hypothermia |
| Ali, 2009 | RCT | KMC | Control | 58 | 56 | LBW infant |  | Mean duration of hospital stay,  Hypothermia,  Sepsis |
| Arya, 2021 | RCT | KMC | Control | 1609 | 1602 | LBW infant | Death between enrollment and 28 days | Mean duration of hospital stay,  Hypothermia,  Suspected sepsis,  Exclusive breastfeeding at discharge,  Exclusive breast-feeding at end of neonatal period |
| Brotherton, 2021 | RCT | early KMC | Control | 138 | 141 | LBW infant | Death between enrollment and 28 days | Mean duration of hospital stay,  Hypothermia,  Exclusive breastfeeding at discharge |
| Chwo, 2002 | RCT | KC | Control | 17 | 17 | premature infant |  | Mean duration of hospital stay |
| Gathwala, 2008 | RCT | KMC | Control | 50 | 50 | LBW infant |  | Mean duration of hospital stay,  Sepsis |
| Hake-brooks, 2008 | RCT | KMC | Control | 36 | 30 | preterm infant |  | Exclusive breastfeeding at discharge |
| Kadam, 2005 | RCT | KMC | Control | 44 | 45 | LBW infant |  | Mean duration of hospital stay,  Hypothermia |
| Lumbanraja, 2016 | RCT | KMC | Control | 20 | 20 | LBW infant |  | Mean duration of hospital stay |
| Mazumder, 2019 | RCT | KMC | Control | 4470 | 3914 | LBW infant | Death between enrollment and 28 days | Exclusive breast-feeding at end of neonatal period |
| Mwendwa, 2012 | RCT | KMC | Control | 85 | 81 | LBW infant |  | Mean duration of hospital stay |
| Roberts, 2000 | RCT | KMC | Control | 16 | 14 | LBW infant |  | Mean duration of hospital stay |
| Sloan, 2008 | RCT | CKMC | Control | 2121 | 2044 | LBW infant | Death between enrollment and 28 days |  |
| Suman, 2008 | RCT | KMC | Control | 103 | 103 | LBW infant |  | Mean duration of hospital stay,  Hypothermia,  Sepsis |
| Tessier, 1998 | RCT | KMC | Control | 246 | 242 | LBW infant |  | Mean duration of hospital stay |
| Walsh, 2020 | RCT | SSC | Control | 21 | 26 | premature infant |  | Hypothermia |
| Worku, 2005 | RCT | KMC | Control | 62 | 61 | LBW infant | Death between enrollment and 28 days |  |

KMC, Kangaroo-Mother Care (including KC, Kangaroo Care; CKMC, Community-Based Kangaroo Mother Care; SSC, skin-to-skin contact); Control, radiant warmers/open cots in warm room, which is considered as standard care.
