# Supplementary material for: The efficacy of Kangaroo-Mother care to the clinical outcomes of LBW and premature infants in the first 28 days: A meta-analysis of randomized clinical trials
Source: Front Pediatr. 2023 Feb 27;11:1067183. doi: 10.3389/fped.2023.1067183 (PMC10008937; doi:10.3389/fped.2023.1067183)
Supplement: Supplementary file 2 [file Table2.docx]

**Supplementary Table 2.** **Risk of bias assessment for each of the included randomized controlled trials.**

| Author, year,  Study (RCT) | Sequence Generation | Allocation Concealment | Blinding | Incomplete outcome data | Selective outcome reporting | Free of other bias |
| --- | --- | --- | --- | --- | --- | --- |
| Acharya, 2014 | low risk | low risk | unclear risk | low risk | low risk | low risk |
| Ali, 2009 | unclear risk | low risk | low risk | low risk | low risk | unclear risk |
| Arya, 2021 | low risk | low risk | low risk | low risk | low risk | low risk |
| Brotherton, 2021 | low risk | high risk | unclear risk | low risk | low risk | low risk |
| Chwo, 2002 | high risk | low risk | low risk | low risk | low risk | unclear risk |
| Gathwala, 2008 | low risk | low risk | unclear risk | low risk | low risk | low risk |
| Hake-brooks, 2008 | low risk | low risk | unclear risk | low risk | low risk | unclear risk |
| Kadam, 2005 | low risk | low risk | low risk | low risk | low risk | low risk |
| Lumbanraja, 2016 | low risk | unclear risk | low risk | low risk | low risk | low risk |
| Mazumder, 2019 | low risk | low risk | low risk | low risk | low risk | low risk |
| Mwendwa, 2012 | low risk | low risk | low risk | low risk | low risk | low risk |
| Roberts, 2000 | low risk | low risk | low risk | unclear risk | low risk | low risk |
| Sloan, 2008 | high risk | high risk | unclear risk | low risk | low risk | unclear risk |
| Suman, 2008 | low risk | low risk | low risk | low risk | low risk | low risk |
| Tessier, 1998 | low risk | low risk | unclear risk | low risk | low risk | low risk |
| Walsh, 2020 | low risk | low risk | low risk | low risk | low risk | low risk |
| Worku, 2005 | low risk | unclear risk | low risk | low risk | low risk | low risk |

The RCTs were assessed by the Cochrane Collaboration’s tool. Risk of bias was assessed as “low risk”, “high risk” or “unclear risk”.
