# Supplementary figures and images for: The efficacy of Kangaroo-Mother care to the clinical outcomes of LBW and premature infants in the first 28 days: A meta-analysis of randomized clinical trials
Source: Front Pediatr. 2023 Feb 27;11:1067183. doi: 10.3389/fped.2023.1067183 (PMC10008937; doi:10.3389/fped.2023.1067183)

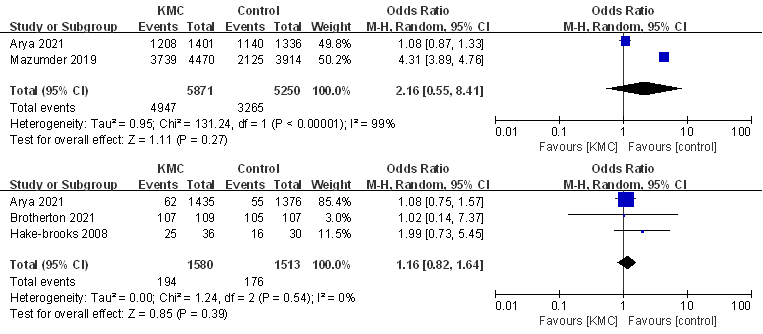

Supplement: Supplementary file 3 [file Image1.tif]

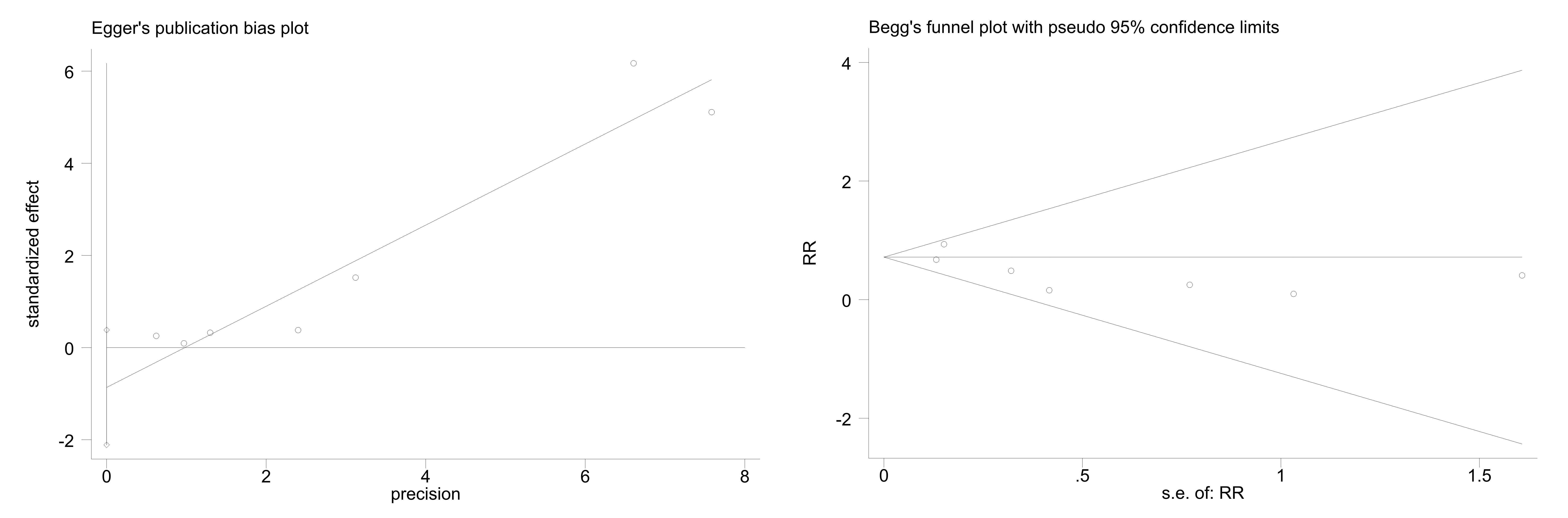

Supplement: Supplementary file 4 [file Image2.tif]

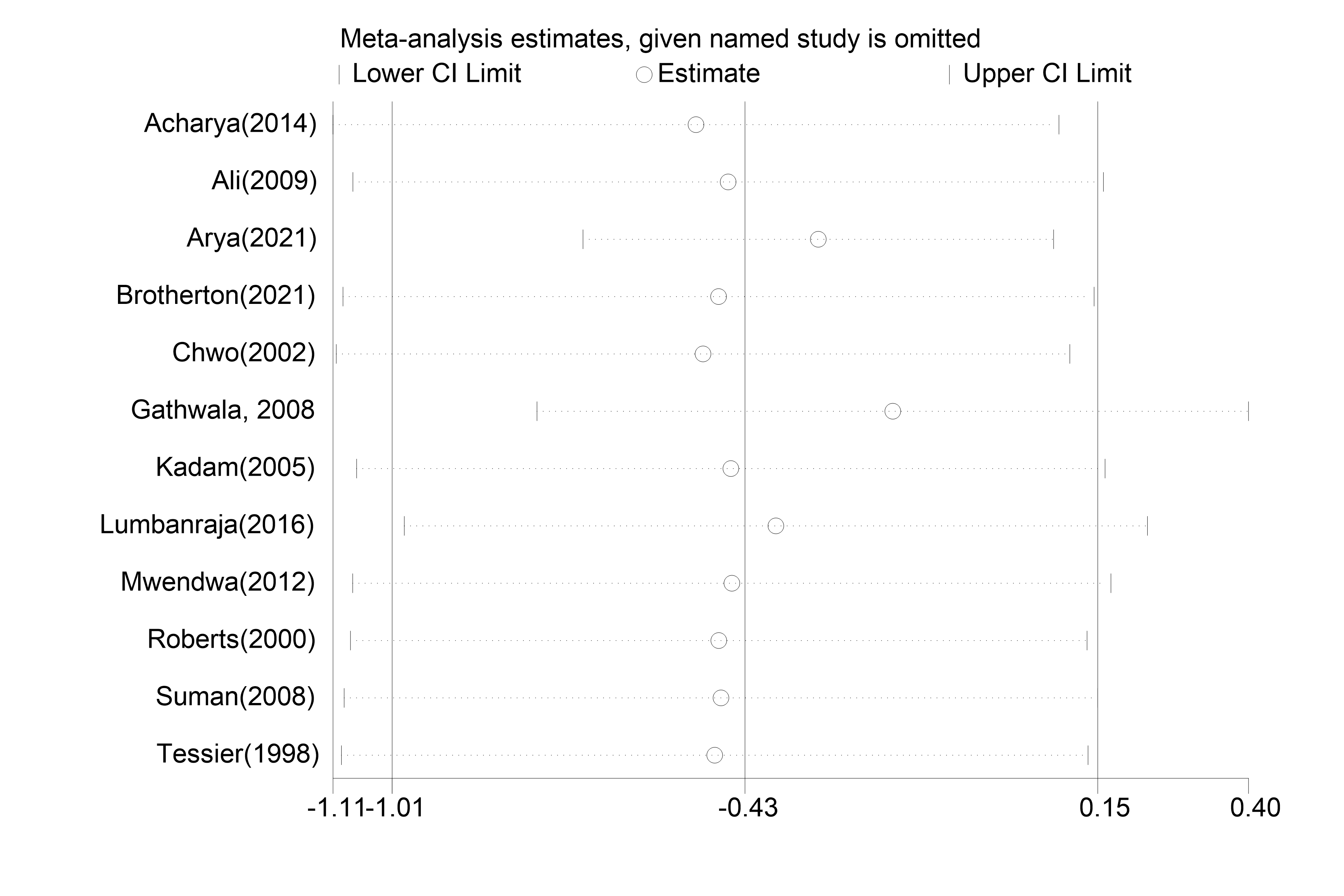

Supplement: Supplementary file 5 [file Image3.tif]
